# Deep CNN-LSTM With Self-Attention Model for Human Activity Recognition Using Wearable Sensor

**DOI:** 10.1109/JTEHM.2022.3177710

**Published:** 2022-05-25

**Authors:** Mst. Alema Khatun, Mohammad Abu Yousuf, Sabbir Ahmed, Md. Zia Uddin, Salem A. Alyami, Samer Al-Ashhab, Hanan F. Akhdar, Asaduzzaman Khan, Akm Azad, Mohammad Ali Moni

**Affiliations:** Institute of Information TechnologyJahangirnagar University 115523 Savar Dhaka 1342 Bangladesh; SINTEF Digital 0373 Oslo Norway; Department of Mathematics and StatisticsFaculty of Science, Imam Mohammad Ibn Saud Islamic University (IMSIU) Riyadh 13318 Saudi Arabia; Department of PhysicsFaculty of Science, Imam Mohammad Ibn Saud Islamic University (IMSIU) Riyadh 13318 Saudi Arabia; School of Health and Rehabilitation SciencesFaculty of Health and Behavioural Sciences, The University of Queensland 1974 Saint Lucia QLD 4072 Australia; Faculty of ScienceEngineering & Technology, Swinburne University of Technology Sydney Parramatta NSW 2150 Australia; ProCan^®^Faculty of Medicine and Health, Children’s Medical Research Institute, The University of Sydney 4334 Westmead NSW 2145 Australia

**Keywords:** Sensors, smartphones, accelerometers, attention, gyroscopes, LSTM

## Abstract

Human Activity Recognition (HAR) systems are devised for continuously observing human behavior - primarily in the fields of environmental compatibility, sports injury detection, senior care, rehabilitation, entertainment, and the surveillance in intelligent home settings. Inertial sensors, e.g., accelerometers, linear acceleration, and gyroscopes are frequently employed for this purpose, which are now compacted into smart devices, e.g., smartphones. Since the use of smartphones is so widespread now-a-days, activity data acquisition for the HAR systems is a pressing need. In this article, we have conducted the smartphone sensor-based raw data collection, namely *H-Activity*, using an Android-OS-based application for accelerometer, gyroscope, and linear acceleration. Furthermore, a hybrid deep learning model is proposed, coupling convolutional neural network and long-short term memory network (CNN-LSTM), empowered by the self-attention algorithm to enhance the predictive capabilities of the system. In addition to our collected dataset (*H-Activity*), the model has been evaluated with some benchmark datasets, e.g., MHEALTH, and UCI-HAR to demonstrate the comparative performance of our model. When compared to other models, the proposed model has an accuracy of 99.93% using our collected *H-Activity* data, and 98.76% and 93.11% using data from MHEALTH and UCI-HAR databases respectively, indicating its efficacy in recognizing human activity recognition. We hope that our developed model could be applicable in the clinical settings and collected data could be useful for further research.

## I. Introduction

Human Activity Recognition (HAR) is a challenge that aims to forecast user activities based on device interactions. It aids people in their daily lives in a variety of ways. Human activity can be detected using two methods: video image recognition and wearable sensors. Through video systems, the camera is used to recognize human behavior. This strategy not only necessitates the installation of costly cameras and infrastructure, but it also creates issues because of the background, lighting, and scale circumstance that make movement detection difficult. In the following method, human activity identification relies on wearable sensors, in particular accelerometers, magnetometers, gyroscopes, linear acceleration and so forth convert motion into identified signals. It adds a new dimension to moving with fewer environmental constraints than the video-based method and provides the privacy of the user [1]. Wearable activity detectors, like the well-known Pedometer, are utilized in various healthcare applications for day-to-day fitness tracking [2], [3]. Despite this, the efficiency of those methods remains debatable [4], and much work is being done to enhance the contribution of inertial sensors for Human Activity Recognition (HAR). Although deep learning techniques for machine learning have recently taken a lot of consideration in the research community, deep learning norms are still underutilized for training time series of inertial sensor data for action detection [3], [5]–[8]. There have been numerous suggestions in the literature [3], [9]–[12] of traditional machine learning and profoundly trained methods for HAR using accelerometers over the last decade. In contrast, real-world HAR systems may fail to distinct to new members and/or situations, resulting in low computational efficiency in practical applications [13], [14]. The accuracy of activity-recognition algorithms can affect by a multitude of reasons, including (i) device position (for example, hand, pocket, or bag), or (ii) differences between sensor brands in terms of sensitivity range and sample frequency. While extensive explore has been completed on the effects of human traits on recognition accuracy, few studies have looked into the effects of position and device features [13], [15]–[18]. Lane *et al.* [17] developed a novel approach to incorporating human aspects. This method improves recognition accuracy by taking advantage of user similarity and weighting training data. Unfortunately, the researchers are unable to replicate the results obtained because the dataset which used for the experiment is not open access, and the authors’ primary focus was on automatic annotation of inertial signals rather than the classification of subject activities. The proposed approach by Lane *et al.* [17] merits further investigation, which stems the research conducted in different disciplines.

Throughout this article, we explored the recognition of different activities i.e., standing/sitting, normal walking, running, and jogging on smartphone devices, and proposed an efficient and seamless combination of Convolutional Neural Network (CNN), Long Short Term Memory (an improvement of Recurrent Neural Network (RNN)) and a self-attention model for representing activity features. During the data collection phase, the use of smartphones is assumed to be unconstrained, and inertial sensors are of acceleration, gyroscope, linear acceleration types. In previous studies, features extracted from Micro-Electro-Mechanical Systems (MEMS) signals revealed that the gyroscope and accelerometer signals contain the most information about human motion because they measure kinematic motion indirectly [19]. CNN and LSTM for robust activity feature extraction are used for the time series convolutions features. The above activities allow for supervised training to finally be carried out for the human activity recognition models. We have collected data with an app, namely ‘sensor data collector’ and we named our dataset is $H-Activity$ dataset. H-Activity and two additional publicly available datasets, namely MHEALTH [20], [21] and UCI-HAR [22] were used for evaluating the proposed method. Smartphones were used to collect sensor data and stored for using in our study. The smartphone sensor data was obtained through experiments with ten participants and collect Standing/sitting, walking, jogging, and running data. These findings suggest that if sensor data is properly calibrated and advanced machine learning techniques like CNN, LSTM (Long Short Term Memory) and transformer learning architecture are used, a smartphone can be a powerful tool for recognizing human activities.

The contributions of this paper are listed as below:

• Our system is the combination of deep learning with self-attention model and wearable sensor-based human activity recognition framework that utilizes various smartphone sensors.
• The proposed method uses only a three-axis accelerometer, gyroscope, and linear acceleration to provide reliable recognition performance, where no other sensors such as Global Positioning System (GPS) or a pressure sensor can perform likewise.
• Creating and evaluating a database (H-Activity): A total of ten subjects data is collected for four activities. Sensors on smartphones are used to collect data. The sensors were put in the user’s right trouser pocket. Each activity was captured at a sampling rate of 10 Hz. Sensors capture a total of 9 attributes for each sample. Triaxial acceleration, gyroscope, and linear acceleration sensors were employed in the right pocket.
• We proposed the deep CNN-LSTM with self-attention model for activity classification problem using our own dataset H-Activity as well as two public datasets; MHEALTH and UCI HAR. Classification accuracy reached up to 99.93% for the H-Activity, and 98.76% and 93.11% for MHEALTH and UCI-HAR respectively.
• The suggested method is used to lessen the dependency on traditional Machine Learning ML) techniques that extract features in a handcrafted process.

This article has the following sections. The literature review, including related work, problem description, motivation, and feasibility analysis, as well as the advantages of the proposed method over existing methods, is described in Section II. Section III discusses the design of the system with H-Activity, MHEALTH and UCI-HAR datasets description, acquisition, and preprocess of the H-Activity dataset. The suggested human activity recognition framework is described in Section IV. Section VI discusses the results after describing the experiments and validation in Section V. Finally, Section VII and section VIII are for discussion and conclusion, respectively.

## II. Literature Review

This section describes existing work, the problem, and the advantages of the proposed work over existing techniques.

### A. Related Work

Activity recognition has been used in a number of situations [23]–[25], like individual authentication [26]–[28], medical examination [29], [30], elderly person wellness monitoring, development of wearable and smartphone based tracking systems, and impersonation attack protection [56].

In the topic of human activity recognition, a significant amount of study has been conducted. The activity data collection methods usually varied of the sensing modality used. For the sake of this paper, we will exclusively explore strategies that make use of Smartphone sensor data. Smartphone sensor-based activity analysis has drawn the attention of researchers due to factors such as availability, affordability, and portability, as it eliminates the need for a sophisticated laboratory setup and expensive equipment.

Activity classification and feature extraction techniques have been studied in previous HAR. Deep learning is the rapidly emerging field which automates the aforementioned techniques. The deep learning technique, which employs numerous layers in the system, identifies ideal characteristics from raw data without the need for human interaction [31]. According to several research, this method can produce very accurate activity classification findings [32]–[34]. However, the application has limitations and challenges. To begin, training a deep learning model necessitates a large amount of data. Second, the model is typically treated as a black box, and the derived characteristics from the multi-layered approach may be difficult to understand [31], making algorithm improvement difficult. In [35] Long Short Term Memory has been used because of its nonlinear properties. The authors proposed a model for predicting green house climate change. Nevertheless, their used sensor collects incorrect data, but the model they proposed performs well with abnormal data. To recognize the upper limb gesture in a rehabilitation setting, the authors in [36] used a fully connected deep learning approach. They compare their model to various machine learning algorithms and show that the proposed fully connected neural network outperforms them in gesture recognition. In addition, the authors of [37] demonstrated that a category-aware gated recurrent unit model for the next POI category recommendation performs better than other baseline methods.

The researchers in [38] are working on human gait analysis for various clinical and pathological trails of patients with stroke, Parkinson’s disease, old stage walking issues, and other neurological disorders. They employ several machine learning techniques that necessitate the services of a feature extraction expert. They proposed utilizing cellular automata to forecast human gait state and ELM to classify it. Human gait analysis is being considered by technology experts as a biometric identity verification method, multi-mode gesture generation, and the creation of human-like robot walking patterns. Semwal *et al.* [39] proposed an optimized feature for gait data categorization based on incremental feature analysis. This study relies entirely on skeleton characteristic data derived from human actions and deep learning models.Gupta *et al.* suggested a hybrid strategy for recognizing human walking behaviors using an ensemble learning method in [40].In [41], Bijalwan *et al.* presented a combination of wearable sensor-based and kinect sensor-based strategies for generating person stepping patterns, as well as constitutive models of the work.

### B. Problem Description, Motivation, and Feasibility Analysis

In this Fourth Industrial Revolution (4IR or Industry 4.0) era, the digital world has a plethora of data, such as mobile sensor data, security data, health data, and so on. Knowledge of artificial intelligence (AI), particularly deep learning (DL), is required to cognitively interpret these data in order to develop smart and automated applications such as elderly health issues and security systems, wearable and phone-based tracking systems, etc. In this field, deep learning algorithms of various types, such as convolutional neural network and long short term memory, are available. Deep learning is capable of efficiently analyzing large amounts of data. We present a comprehensive overview of deep learning algorithms that can be used to recognize human activity in this paper. Physical phenomena such as unbalance, stumbling, recurrent falls, staggering, and freezing in daily human movements are referred to as activity disorders. It can be caused by one of two factors: neurological or non-neurological. It’s fairly common in adults in their forties and fifties, as well as those over the age of 80. Human activity recognition and classification, among other things, aid in the identification of neurological problem patients, hemiparetic patients, and the examination of sports-person activity patterns [38]. It is a sensor-based analysis technique that employs a variety of sensors to capture human activity or movement patterns. The use of smartphone sensors reduces the overall cost of the system.

### C. Advantages of the Suggested Method Over Existing Methods

The following are some of the advantages of the suggested approach over existing techniques: In contrast to present systems [42], which only recognize walking as an activity, the suggested concept recognizes four different activities. The proposed method is more accurate than the existing method. H-Activity is the name of the data set we have constructed. The suggested study provides a basic framework for activity recognition as well as activities that are well suited for human activity analysis in clinical trials.

## III. System Design

**FIGURE 1.**
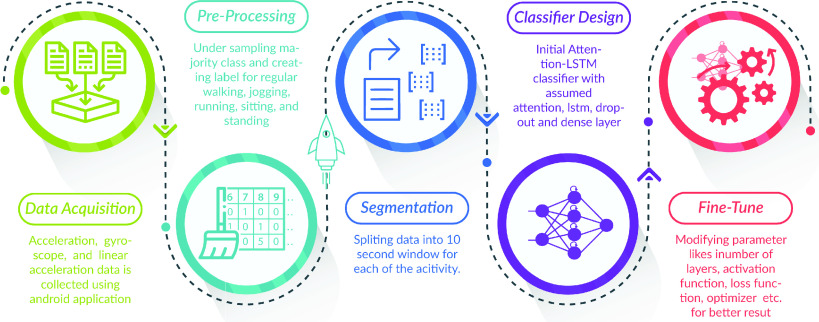
The schematic diagram of our proposed workflow. Raw data are firstly acquired from sensors. After preprocessing, segments of data are extracted (known as *Segmentation*) and a classifier is designed. Fine tuning is used to adjust the hyper-parameters. The classifier is then trained and evaluated using those features (known as *Classification*).

The objective of this research is to develop a model that can predict human activities including walking, standing/sitting, jogging, running, and so on. In the development of our human activity recognition system, a systematic workflow incorporating sensor engineering, data processing, and deep learning techniques is usually followed. This section discussed about the sensors, dataset and processing criteria that were used in the experiments. Figure 1 schematizes this approach, which consists of the following steps:

### A. Description and Validation of the Dataset

Several wearable sensors-based datasets are available to detect human activity. However, the majority of data are gathered using a variety of sampling rates, sensor counts, sensor placements, and recorded activity counts. Using H-Activity as well as two commonly used datasets, including MHEALTH (mobile health) [20], [21] and UCI-HAR dataset [22], the suggested technique and current approaches provided in the study were experimented and verified. These datasets are organized at various sample rates, the number of sensors and the number of activities recorded. A few of these datasets are well-balanced, while others are significantly unbalanced. We have compared a classification method across different datasets. Although these datasets have various frequencies and activities, our goal is to establish the novelty of the same LSTM-CNN classifier that can produce satisfactory results in different circumstances. One way to compare the data is to make them similar, as done in [43]. Though we can only change the data of proposed datasets, since we have the complete data for each activity, we can divide them using separate methods such as transformation. However, it is not easily attainable for the remaining datasets.

**FIGURE 2.**
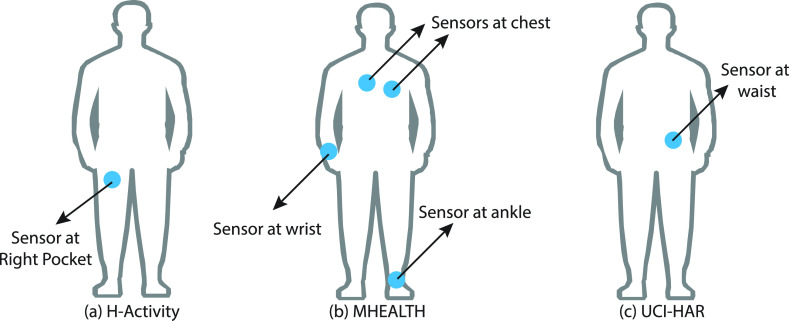
Inertial sensor placement in various datasets (a) H-Activity (b) MHEALTH (c) UCI-HAR.

A brief summary of the H-Activity, MHEALTH, and UCI-HAR that were used in the proposed study is discussed in Table 1. Figure 2 shows the sensor placement on subject’s body. The relevant sections provide a quick overview of the mentioned datasets:

**TABLE 1:** A Concise Narration of the H-Activity, MHEALTH, and UCI-HAR Datasets Used in the Proposed Experiment

Dataset	Type of sensor and location	Sampling Freq.	Samples	Volunteers	Placement of the sensor	Type of activities
H-Activity	Phone based accelerometer, gyroscope, and linear acceleration	10 Hz.	48920	10	Smartphone in trousers right pocket	4 activities. Sitting/standing, walking, jogging, and running
MHEALTH	accelerometer, gyroscope, and magnetometer	50 Hz	120	10	Sensors were implanted on the participant’s chest, right wrist, and left ankle.	12 physical activities such as standing, sitting, lying, walking, climbing, cycling, jogging, running jumping.
UCI-HAR	Phone based accelerometer, and gyroscope	50 Hz	10299	30	Smartphone in Waist	6 activities such as: Walking, going upstairs/downstairs, sitting, standing, and lying

#### 1) H-Activity

The H-Activity dataset was gathered from ten volunteers between the ages of 20 and 30, who kept their smartphones in their right trouser pockets. All of the volunteers were participating in 4 different physical activities. For instance: sitting/standing, walking, jogging, and running.

#### 2) Publicly Available Data: MHEALTH and UCI-HAR

To validate our collected dataset, we use the previously available MHEALTh and UCI—HAR datasets as a benchmark for making comparisons with our collected data.

Sensors placed at the left, chest, and right handles of 10 volunteers collected body signals and emergent indicators (swing rate, acceleration, magnet field direction), while performing 12 physical activities, such as Sitting and relaxing (Sit), Climbing stairs (CS), Walking (Walk), Standing still (Std), Waist bends forward (WBF), Climbing stairs (CS), Frontal elevation of arms (FEA), Jogging (Jog), Knees bending (crouching) (KB), Cycling (Cycl.), Lying down (Lay), Jump front & back (JFB), Running (Run). A sample rate of 50 Hz was used to record all the actions.

Dataset UCI-HAR is made up of data collected by 30 people. The ages of the participants range from 19 to 48 years old. Walking (Walk), going upstairs (Up)/downstairs (Down), sitting (Sit), standing (Std), and lying (Lay) are among the six actions performed by the participants. A *Samsung Galaxy S II* smartphone was attached to the waist for collecting accelerometer and gyroscope data. Fixed segmentation of the data with 50% overlap was employed for this data collection. A butterworth low-pass filter was employed to distinguish the components of gravitation and body motion of the sensor data into the acceleration of body and gravity. The remaining 30% of the participants were utilized for testing and the rest 70% were employed for training. During testing, this data splitting was leveraged to generate new, previously unseen data. This dataset contains 10,299 samples.

### B. Acquisition and Preprocessing of H-Activity Dataset

In order to collect the time-series H-Activity data, we used an Android-OS-based application, namely *Sensors Data Collector* to select the inertial sensors, i.e., acceleration, gyroscope, and linear acceleration. Further details of this application can be found in reference [44]. This data collection app has a lot of customization options available and the user has the option of selecting among Sitting/Standing, Walking, Jogging, and Running. In addition, the user can customize the sensors readings (which are by default set to one hour) and the data capture speed (which is set to fast). Furthermore, participants have the option of selecting which sensors will collect data, for example, a gyroscope, an accelerometer or a linear acceleration.

**FIGURE 3.**
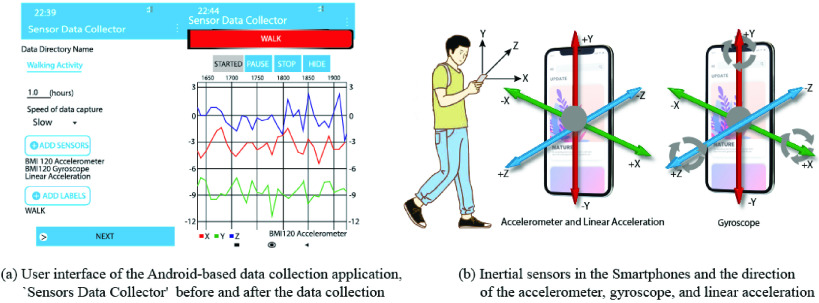
Representation of (a) user interface of the Android-based data collection application, ‘Sensors Data Collector’ before and after the data collection (b) inertial sensors in the smartphones and the direction of the accelerometer, gyroscope, and linear acceleration.

The data collection program, ‘Sensors Data Collector’ shown in Figure 3(a) is used to gather information from 3 sensors: accelerometer, gyroscope, and linear acceleration during these activities. The way in which machine learning algorithms function was not to read a huge quantity of data at once, so that each entry in the H-Activity dataset was done with 10-sec segments, as recommended in [45]. The app captured raw data from each line of sensors, each of which has 3 axes: *X, Y,* and ${Z}$. The major problem in this section is that the Android platform does not allow data from a sensor to be read at a particular moment. In practice, the data can only be read when a shift in the sensor reading is detected. Another issue was noise filtering. The data was not subjected to noise cancellation. To avoid inaccurate labeling, topics have to come to a complete halt, waiting for a couple of seconds before proceeding to the next [46] before each action. Accelerometer, gyroscopic and linear data from the start and stop periods of the activity were used as the name of the activity. The sampling rate was 10Hz, which means one sample was taken every 1 second. A total of 48,920 three-dimensional acceleration, gyroscope, and linear acceleration samples were collected.

Both an accelerometer and a linear acceleration sensor calculate the force applied to a system in ‘meters per second’ on all 3 physical axes (*X, Y,* and ${Z}$). The difference between the two is that the accelerometer includes the force of gravity, whereas the linear acceleration does not. The data was unbalanced, particularly when walking normally, for which ‘class balancing (i.e., Under-sampling or oversampling) the data set was required. Table 2 shows that during normal walking, the linear acceleration and gyroscope only yielded total 12,233 and 12,247 data, respectively, while the accelerometer gave 77,867 data.

**TABLE 2:** Data Distribution with Different Human Activity

	Linear Acceleration	Gyroscope	Accelerometer
(Sitting/Standing)	12,119	12,423	12,865
Normal Walking	12,233	12,247	77,867
Jogging	8,595	9,599	13,821
Running	8,582	9,162	18,182

In the proposed method, the nine features, namely accelerometer, gyroscope, and linear acceleration in the x,y, and z axes, are used as input to the learning algorithm. Each sample is labeled as the data is processed. Under and Over Sampling, Linear interpolation and data segmentation are performed, followed by one-hot vectorization.

#### 1) Under-Sampling and Over-Sampling

Each of the three sensor data represents the same time duration, though it contains a different amount of data for a similar time stamp. Since each sensor has a different sampling rate and vendors, the number of data points varies for accelerometer, linear acceleration and gyroscope. To our observation, an accelerometer generates more data than a gyroscope and linear acceleration. One solution to the issue of class imbalance is to resample the training dataset at random. Under-sampling (deleting samples from the majority class) and over-sampling (duplicating instances from the minority class) are the two key ways to randomly resample an imbalanced dataset. Under-sampling is generally beneficial, while random oversampling is not. SMOTE (Synthetic Minority Oversampling Technique) is a widely used oversampling technique that was developed to enhance random oversampling. To overcome the problem of data imbalance, it was then over-sampled using minimum and maximum values. Table 3 illustrates the distribution of data with different activities after sampling.

**TABLE 3:** Data Distribution with Different Human Activity After Sampling

	Linear Acceleration	Gyroscope	Accelerometer
(Sitting/Standing)	12,110	12,110	12,110
Normal Walking	12,230	12,230	12,230
Jogging	8,590	8,590	8,590
Running	8,580	8,580	8,580

#### 2) Inertial Sensors in the Smartphone

An Inertial Measurement Unit (IMU) is a digital gadget that assesses or calculates and states a specific force, angular rate, and, in some cases, magnetic fields surrounding the body using an accelerometer and gyroscope combination. Currently, most smartphones support all of these sensors. The inertial dynamics are measured in three directions along the x, y, and ${z}$ axes, as shown in Figure 3(b). The three-way accelerations change linearly with the smartphone velocity in 3D space and depict the movement of smartphone users. Smartphone’s angular speed is collected by the three-axis gyroscope, as it spins in space and can also be used to define the user movement. Smartphones of various brands such as *Samsung, Xiaomi*, and *Huawei*, all running the Android operating system, have been used in this study. The smartphone is turned on when a user walks and the spinning occurs according to the user’s movement. These data are each deemed unique so that we can collect them for Guide Dynamics as source data. The smartphone can handle the accelerometer, gyroscope and linear acceleration with hardware synchronization. If intermediate data are recorded via the Android interface, it can affect a certain level of synchronization. But it is bearable and has minimal impact on our system of recognition of activity. In this investigation, the accelerometer is employed for the x-axis, y-axis and z-axis three times. The body movement and gravity combine each time series with linear acceleration. We’re mostly interested in gathering data on three types of oriental motions (horizontal, vertical, and backward/forward with relation to the x, y, and z-axes). Figure 3(b) shows an example of tri-axial data illustrating a user conducting walking.

#### 3) Linear Interpolation

We acquired the H-Activity dataset practically by using the smartphone’s sensors. The smartphone is placed in the right pockets of subjects. As a result, some used data has been lost during the compilation process, which is typically represented by NaN or 0. This problem can be overcome by using the linear interpolation algorithm and filling the missing value [47]. We used the interpolation algorithm to deal with the problem.

#### 4) Segmentation

In this article, a model for the recognition of human activity was introduced. A data sequence is used to create the model input. The sequence is derived from a raw sensor’s short time series data and data was collected continuously throughout the data collection process. To retain the temporal relationship between data points, a sliding operation for segmentation was used, which had the size of 10 in length. Each window of readings is a 9-feature data point and the size of the data is 6 MB.

#### 5) Class Relabeling and One-Hot Encoding

**FIGURE 4.**
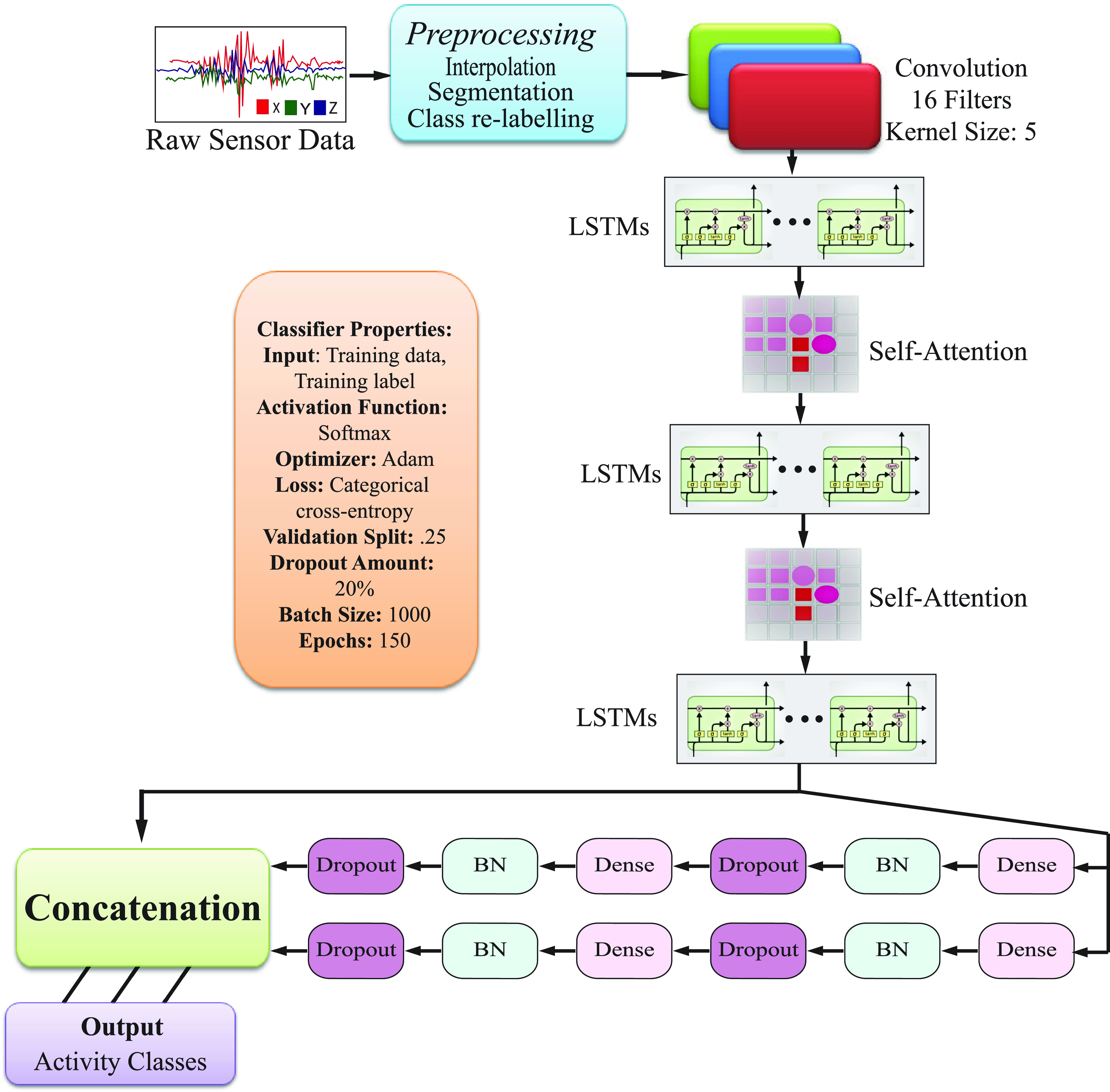
The proposed Deep CNN-LSTM with self-attention model architecture for human activity recognition.

The output labels have also been converted to one-hot encoded labels. As discussed in the H-Activity dataset description section, we coded our activity windows into four unique labels. These are: ‘0’ for sedentary (stand + sitting), ‘1’ for normal walking, ‘2’ for jogging, and ‘3’ for running. The steps of pre-processing are also summarized in Figure 4.

## IV. Proposed Human Activity Recognition Architecture

The proposed HAR model architecture, which is based on CNN-LSTM with a self-attention model, is used to classify smartphone users’ activities. In this study, the framework allows for the collection of sensor data from a smartphone sensor. Figure 4 depicts the structure of the proposed CNN-LSTM with the self-attention model. It consists of nineteen layers. Preprocessing of raw sensor data is described in sections III(B).

The pre-processed input data is first fed into a 16-filter convolutional neural network, which is then followed by a batch normalization layer and a dropout layer with a 20% rate. The output is then fed into a 64-neuron two-layer LSTM, which is commonly used to extract temporal information. Second, the output of the LSTM layer has been transmitted into the self-attention layer. The attention layer is primarily used to focus on a particular network layer. The second LSTM, attention, and dropout layer employed the same number of neurons and dropout rate as the previous step. The activation is accomplished through the use of a’sigmoid’ feature. Finally, an output layer (a dense layer with a’softmax’ classifier) is used to obtain the model’s output. The Adam optimizer outperformed the other three types of optimizers (i.e., Adagrad, RMSprop, and SGD). All of the parameters, as well as binary and categorical cross-entropy, were tested on a trial-and-error basis, and the best one was chosen.

### A. Neural Network for Human Activity Recognition: Model Implementation

The human activity recognition network, as shown in Figure 4, is made up of a CNN, LSTM, attention, and a dense network. The activities of the subject are recognized using a dense network, which performs the function of a classifier using the residual concatenation for classification, followed by CNN, LSTM and the attention model.

#### 1) Convolutional Layer

Figure 4 depicts the proposed CNN-LSTM framework with self-attention model, which employs CNN layers to automatically extort characteristics from facts, as well as combined with LSTMs and an attention layer to aid sequence forecasting. CNN-LSTMs with self-attention are employed in the solution of visual time series forecasting problems and the generation of textual records from image series. This construction is relevant for problems that require temporal output generation or that involve temporal input structures. This paper proposes a deep CNN-LSTM with self-attention model to improve recognition performance.

\begin{equation*} a_{i,j} = {f\left({\sum _{m=1}^{M}\sum _{n=1}^{N}w_{m,n}.x_{i+m,j+n}+b}\right)}\tag{1}\end{equation*}

where $a_{i,j}$ is the respective activation, $f$ is the non-linear activation function, $W_{m,n}$ represents the weight matrix of convolution kernel as $\text{m}\times \text{n}$, $X_{i+m,j+n}$ denotes the upper neuron activation connected to the neuron $(i,j)$, and $b$ is the bias term.

In our experiment, rectified linear units (ReLU) are used by convolutional layers in the calculation of the feature maps, where the non-linear function is denoted like the following:

\begin{equation*} \sigma (x) = max(0,x)\tag{2}\end{equation*}

In general, it is examined that the more convolution kernels are used, the more hidden features of the input samples could be extracted [48]. The CNN-LSTM with self-attention model have one convolutional layer. 16 kernels are used for extraction of features in this convolution layer, with a size of $1\times 5$ for every convolutional kernel.

#### 2) LSTM Network Structure

Nowadays, LSTM networks [49] perform admirably across a wide range of temporal schemes. The LSTM is a type of Recurrent Neural Network (RNN) that is growing in popularity. RNNs can estimate the present time output relied on previous knowledge in the DL approach. However, due to the disintegrating gradient problem, [50] states that RNN systems can only identify data for a short period of time. Gradients will be submerged if they are not allowed to flow deeply while using the deep learning back-propagation strategy. To address the challenge of long-term dependency, [51] proposed a novel neuron to the RNN group called LSTM.

To extract the temporal aspects in the sequence data more effectively, the input data is first passed through two-layer of LSTM in this paper. There are 64 memory cells in the LSTM layer. The following formula is used to manipulate the action of each LSTM unit by sending various inputs to different gates, such as input, gates, and output gates.

\begin{equation*} h_{t} = {\sigma (w_{i,h}.x_{t}+w_{h,h}.h_{t-1}+b)}\tag{3}\end{equation*}

where $h_{t}$ and $h_{t-1}$ signify activation at time t and t-1, correspondingly, $w_{i,h}$ is the input-hidden layer weight matrix, $w_{h,h}$ is the hidden-hidden layer weight matrix, b is the bias, and a non linear activation function is symbolized by the $\sigma $.

## V. Experiments and Evaluation

### A. Experimental Setup

For a recurrent network, the size of the network depends on the availability of the GPU memory that is used and on the users’ duration of the training. The size of the GPU RAM should be larger to train a larger CNN and RNN. As an example, LeNet5 training requiring 1GB of GPU RAM can be considered. In this article, the experiment is run on a computer with an Intel Core i5 processor, 8 GB of RAM. In terms of software, the Google COLAB server is used to compile the experimental analyses. We have used Tensorflow as a python library for conducting deep neural network training, tensor operations and parameter inference with automatic differentiation. Otherwise, for other array operations, Numpy is used, while matplotlib and seaborn are used for data visualization.

### B. Evaluation Measures

When collecting data on human action in natural settings, class-imbalances in data categories are common [52], and in this article, our data sets are not different from that aspect. The results will achieve high accuracy if the classifier predicts each instance as a majority class and uses the overall classification accuracy to assess the model output. Therefore, the overall classification accuracy is not a good indicator of the model performance evaluation. The F-measure (F1 score) considers both false positives and false negatives, and it incorporates two metrics based on the total number of correctly recognized samples, known as ‘precision’ and ‘recall’. Next, we briefly describe the evaluation criteria that are used in this study:

#### 1) Precision

The ratio of correctly predicted positive observations to total predicted positive observations is known as precision.

\begin{equation*} Precision=\frac {T_{P}}{T_{P}+{F_{P}}}\tag{4}\end{equation*}

In Eq 4 $T_{P}$ and $F_{P}$ denote true positive and false positive respectively.

#### 2) Recall(Sensitivity)

The ratio of correctly expected positive observations to all observations in the actual class is known as recall.

\begin{equation*} Recall (Sensitivity)=\frac {T_{P}}{T_{P}+{F_{N}}}\tag{5}\end{equation*}

In Eq 5, the $T_{P}$ and $F_{P}$ denotes true positive and false positive, respectfully.

#### 3) F1 Score

It is a harmonic average of the ‘Precision’ and ‘Recall’ values. Therefore, this score considers both false positives and false negatives to higher in order to get a higher F1-score. Although it is not as intuitive as accuracy, F1 is generally more useful than accuracy, particularly when the class distribution is uneven.

\begin{equation*} F1 ~Score=\frac {2*{Precision}*Recall}{Precision+{Recall}}\tag{6}\end{equation*}

## VI. Experimental Result Analysis

To obtain the final model with the best results, four different models were designed and tested. As a result, a number of experiments have been carried out in order to fine-tune the parameters. Different input and output sizes, as well as the Convolutional layer, LSTM layer, attention layer, number of dense layers, and dense layer parameters, all were taken into account during this model selection process. Each model has been trained for a total of 150 epochs. Normally, as training epochs increase, DL models accuracy increases and loss (of the cost-function) decreases. Since the proposed model has been converged in a steady accuracy after 150 epochs, only 150 epochs have been used in both for training and validation. A detailed description of the parameter settings for four different models and the optimized model selection process is outlined below.

### A. Hyper Parameter Selection

For selecting the best model with optimized hyper-parameter settings, we’ve designed and tested four different architectures. In the first model, namely *M1*, we used a $10\times 9$ input matrix with two LSTM, two attention models, and two batch normalization, four dropouts, and three dense layers. Then, for each of the two LSTM layers, a total of 64 neurons were used. Furthermore, the output of the last LSTM layer becomes 128, which is the input of two different dense models. These dense networks are the combination of dense, batch normalization, and dropout layer, where all the inputs and outputs of the first dense model have a total of 64 neurons and in the second dense model, only the first input is 128, and the rest of the layers have 64 neurons as input and output.

In the second model, namely *M2*, we have used a $10\times 9$ input matrix with three LSTM, batch normalization, dropout, and dense layer. Then, for each of the three LSTM layers, a total of 32 neurons were used. Furthermore, the output of the last LSTM layer becomes 128 (the input data of the first batch normalization layer). The inputs and outputs of the second and third batch normalization layers are $256\times 256$ and $128\times 128$, respectively.

Next, in the third model, namely *M3*, We used a $10\times 9$ input matrix with two LSTM, three batch normalization, three dropouts, and three dense layers for the third model, namely *M3*. Then, a total of 32 neurons were used for each of the two LSTM layers. Furthermore, the output of the last LSTM layer becomes 128 (the input data of the first batch normalization layer). The inputs and outputs of the second and third batch normalization layers are $256\times 256$ and $128\times 128$, respectively.

And finally, in the fourth model, namely *M4*, which is also our proposed model, we have used a $10\times 9$ input matrix with one CNN model, three LSTM, two attention models, and three batch normalization, five dropouts, and two dense layers. Then, for each of the first two LSTM layers, 64 neurons were used, and the final LSTM layer had 128 neurons. Furthermore, the output of the final LSTM layer was 384, and served as the input to two separate dense models. These dense networks were a mixture of dense, batch normalization, and dropout layers, with all inputs and outputs in the first dense model, totalling 320 neurons, and only the first input in the second dense model totalling 512, with the rest of the layers having 64 neurons as input and output. The dropout layer is used to drop the layers that aren’t required. The number of trainable parameters for these models was 152 902, 156 292, 197 508, and 633,188 respectively.

**FIGURE 5.**
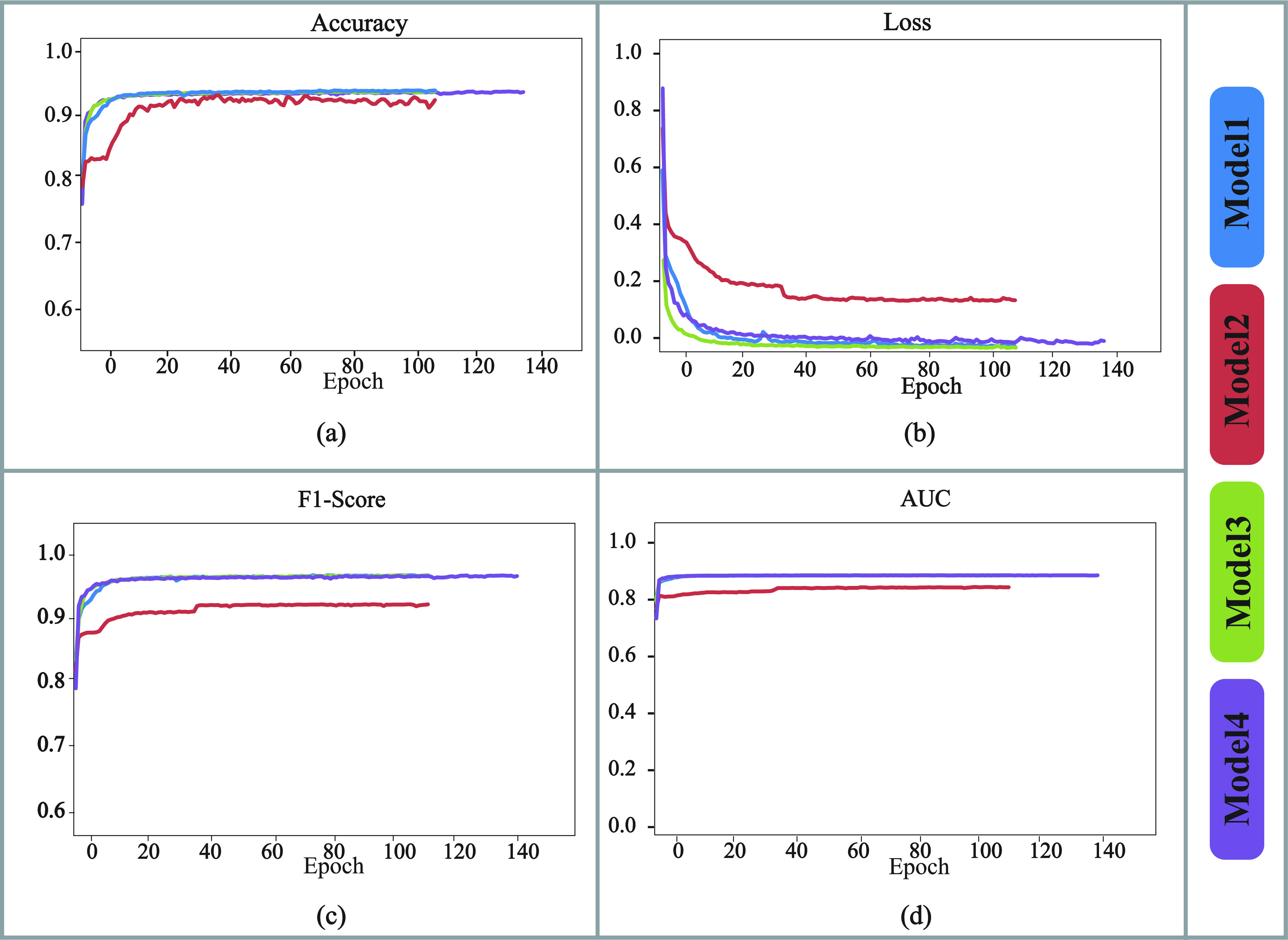
Graphical representation of training (a) Accuracy, (b) losses, (c) F1-Scores, and (d) AUCs of M1, M2, M3, and M4, respectively.

**FIGURE 6.**
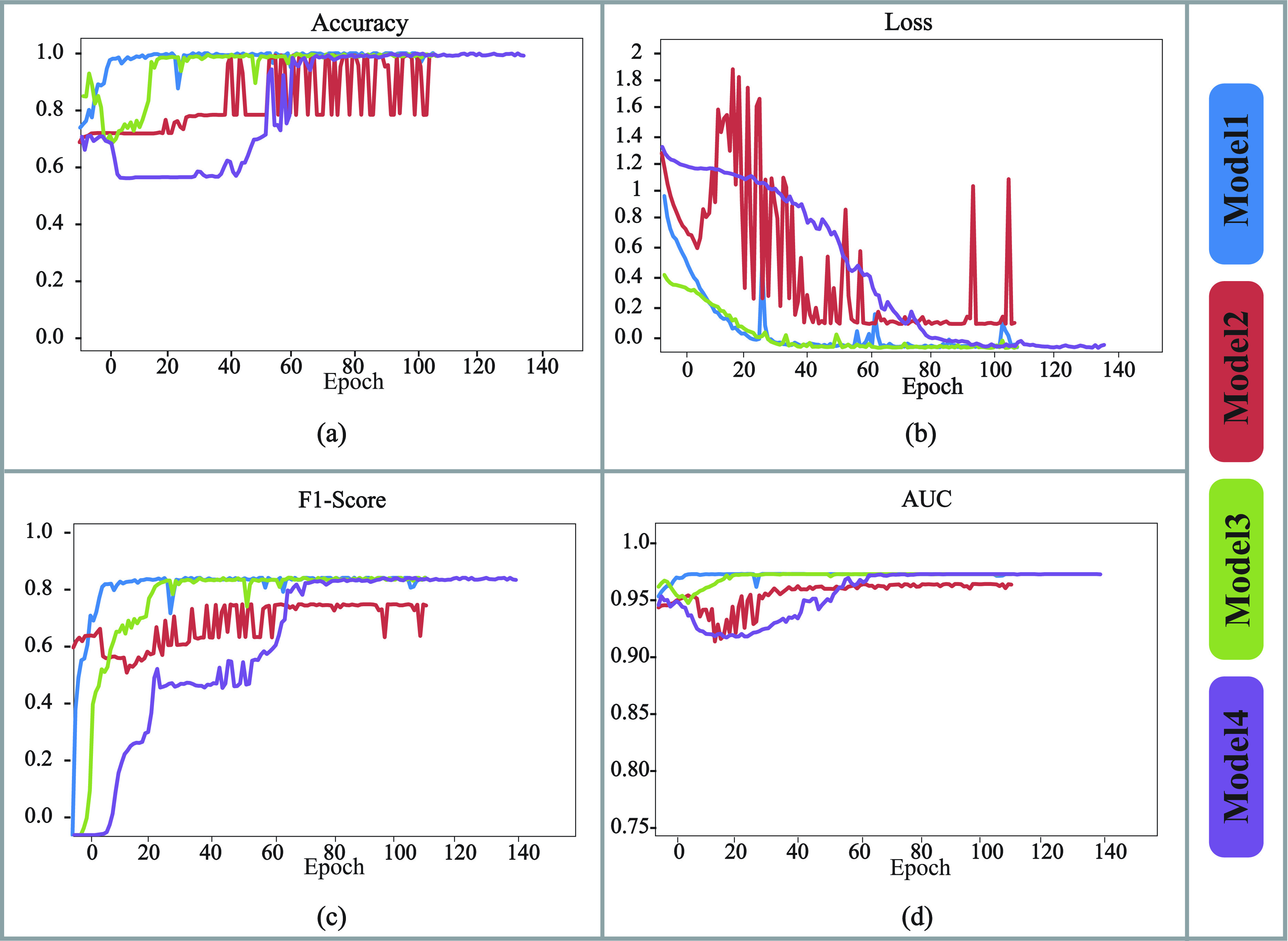
Graphical representation of validation (a) Accuracy, (b) losses, (c) F1-Scores, and (d) AUCs of M1, M2, M3, and M4, respectively.

For each of these four models, the final output was a one-dimensional vector after applying the pixel operations of $320\times 4$, $128\times 4$, $128\times 4$, and $512\times 4$ dimensions, respectively. Model architectures of these four models are outlined in Table 4. The comparative performances of these four models, regarding their training and validation accuracy, Area Under the ROC Curve (AUC), F1 score and Loss are shown in Figure 5 and Figure 6, respectively.

**TABLE 4:** Architectures of Different Models Experimented for Finding the Optimized One

Model	Total Layers	Layer Types	Total Parameters	Trainable Parameters	Non Trainable Parameters
Model M1	15	LSTM, Attention, Dense, Dropout, BN, Concatenate	152,902	152,518	348
Model M2	12	LSTM, Dropout, BN, Dense	197,508	196,484	1024
Model M3	11	LSTM, Dropout, BN, Dense	156,292	155,268	1024
Model M4 (Proposed)	19	CNN, LSTM, Attention, Dense, Dropout, BN, Concatenate	634,052	633,188	864

While finding out the best performing one among these four models, we observed the above performances, where M4 demonstrated the best performance among all others overall. Thus M4 was chosen as the final model for any further analysis. Moreover, M4’s optimal hyper-parameter settings are described in Table 5, which were trained for a total of 150 epochs and corresponding Loss, recall, precision and F1 score have been determined for each epoch of training and testing. Finally, the average validation accuracy for all epochs was achieved as 0.991452719, while the loss was 0.043110911, and F1 score was 0.99182258.

**TABLE 5:** Setting of Hyper Parameter of the Proposed Deep CNN-LSTM With Self-Attention Model

Hyper Parameter	Setting
Input Size	10*9
Epochs	150
Batch Size	1000
Activation Function	Sigmoid
Batch Normalization Axis	−1
LSTM Dropout Rate	0.1
Number of Hidden Layer	2
Output Activation Function	Softmax
Optimizer	Adam
Loss	Categorical Cross Entropy

When predictions were made for the test data set, we presented the confusion matrix plot in Table 678 to put our model performance in perspective. The actual class (Target Class) is defined by the rows in the confusion matrix, while the predicted class is indicated by the columns (Output Class). The uncertainty matrix diagonal cells correspond to correctly classified observations (TP, True positives and TN, True Negatives). In our research data set, there are 4,888 instances of correctly classified for different activities. The findings that were incorrectly labelled (FP, False Positives and FN, False Negatives) are represented by the off-diagonal cells.

All networks are trained with 150 training epochs, where a different number of epochs were used, and we observed that after 150 epoch, the results were repeated, so 150 epoch was finalized. After applying numerous amounts of data as an experiment, we use 25% of the whole dataset for testing.

### B. Model Efficiency As a Result of Hyper-Parameters: Influence Of the Optimizer

**FIGURE 7.**
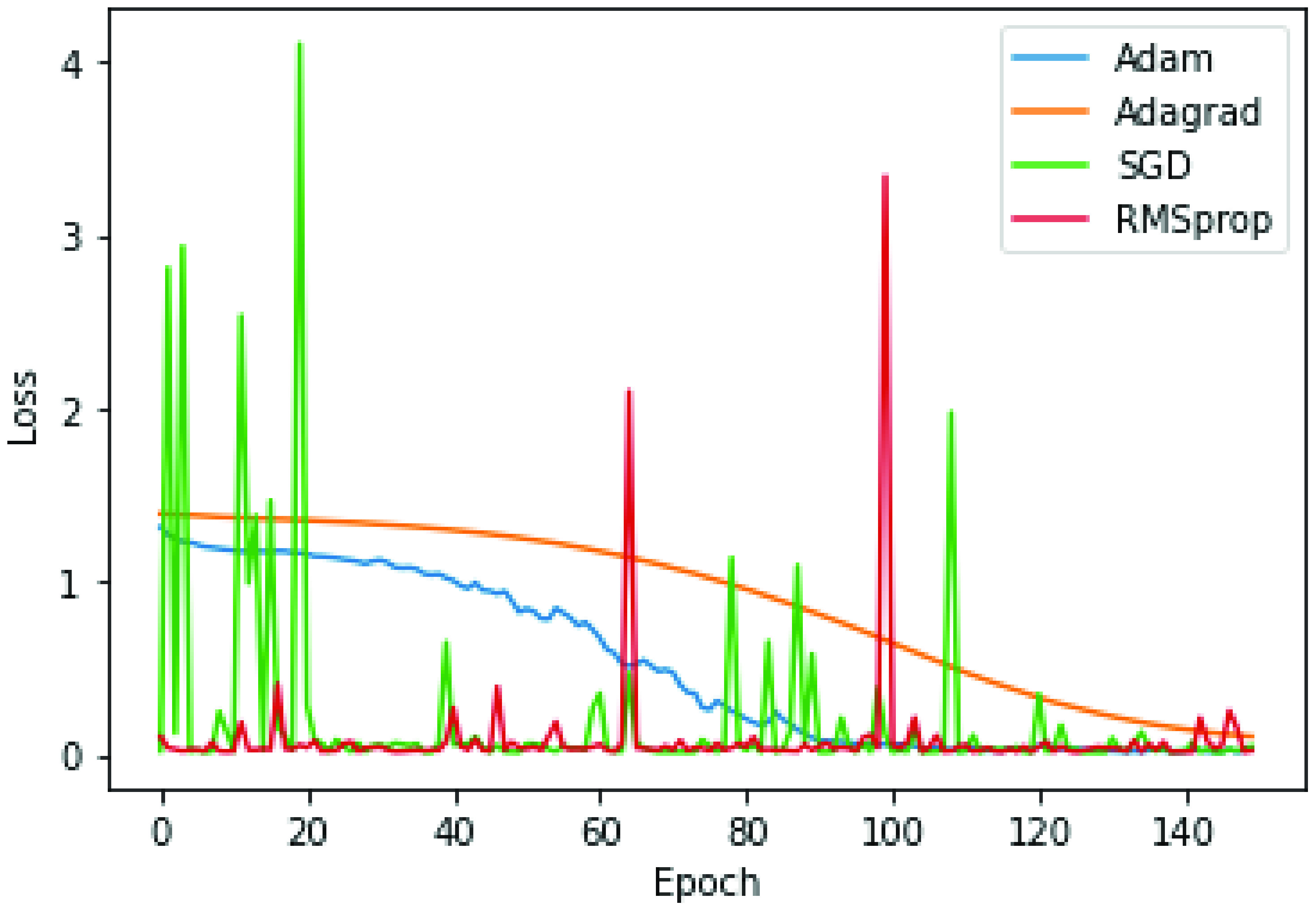
Influence of the optimizer on model performance during training.

The optimizer adjusts and analyses network settings impacting model training and performance to approximate the optimal benefit while decreasing the loss function. As a result, selecting an appropriate optimizer for deep model training is critical. Various well-known optimizers, such as Adam, Adagrad, SGD, and RMSprop, were analytically examined, as shown in Figure 7. It is observed that the Adam optimizer appears to have the greatest effect on model efficiency, with the gradient descent curve fluctuation being the most stable. Hence, when training the CNN-LSTM with self-attention model, Adam was used as the optimizer.

### C. Evaluation on H-Activity, MHEALTH, and UCI-HAR Datasets

H-Activity, MHEALTH, and UCI-HARt were utilized for testing to fully validate the performance of the suggested model. Tables 6, 7 and 8 demonstrate the confusion matrices of classification that were produced when the H-Activity, MHEALTH, and UCI-HAR datasets were predicted for the model. There were 4888 occurrences properly categorized for the H-Activity data set and the total accuracy was 99.93%. The precision and recall were between 99% - 100%. Between jogging and running, there was rather weak differentiation. The principal reason is that from the point of view of motion sensors, the two activities are nearly identical. When the model was subjected to the test set including around 330978 new instances, the overall accuracy of the dataset MHEALTH (it contains 12 activities) reached 98.76%. There were 2714 properly categorized occurrences for the UCI-HAR data set, with total accuracy reaching 93.11%. Between sitting and standing there was rather low discrimination like H-Activity dataset. Recall and accuracy were in the 80% by 100% range. The major explanation for this might be that the two activities are comparable in terms of motion sensors. Deeper information is difficult to extract with simple acceleration, linear acceleration, and angular velocity data.

**TABLE 6:** H-Activity Classification Confusion Matrix

		Predicted						
Activities		Sitting/Standing	Walking	Jogging	Running	Recall(%)
Actual	Sitting/Standing	1222	1	0	0	99.92
	Walking	0	1223	0	0	100
	Jogging	0	1	1220	2	99.75
	Running	0	0	0	1223	100
	Precision (%)	100	99.83	100	99.83	99.93

**TABLE 7:** UCI-HAR Datasets Classification Confusion Matrix

		Predicted						
Activities		Walk	Up	Down	Sit	Std	Lay	Recall(%)
Actual	Walk	474	7	15	0	0	0	93.55
	Up	0	456	15	0	0	0	98.30
	Down	0	9	411	0	0	0	96.67
	Sit	0	22	0	396	71	2	74.95
	Std	0	49	0	99	440	0	82.70
	Lay	0	0	0	0	0	537	100
	Precision (%)	98.93	93.34	93.54	80.00	81.33	98.17	93.11

**TABLE 8:** MHEALTH Datasets Classification Confusion Matrix

		Predicted														
Activities		Std	Sit	Lay	Walk	CS	WBF	FEA	KB	Cycl.	Jog	Run	JFB	Recall(%)
Actual	Std	27632	0	0	0	0	3077	0	20	0	0	0	0	19.90
	Sit	0	30717	0	0	0	1	0	2	0	0	0	0	42.79
	Lay	0	0	30720	0	0	0	0	0	0	0	0	0	100
	Walk	0	0	0	30312	190	78	0	136	0	0	3	1	78.79
	CS	0	18	0	193	28920	757	5	820	4	0	0	3	83.80
	WBF	1	1	0	0	9	28279	22	3	0	0	0	0	94.40
	FEA	37	136	0	4	0	1648	27227	388	1	0	0	0	76.13
	KB	1	3	0	0	32	1473	181	27647	0	0	0	0	71.86
	Cycl.	0	0	0	1	27	21	0	20	30647	0	0	4	83.83
	Jog	0	4	0	39	33	0	8	7	3	30157	243	226	90.11
	Run	0	0	0	14	7	0	0	1	4	1971	28486	237	72.46
	JFB	0	0	0	10	31	0	1	11	6	26	13	10244	97.70
	Precision (%)	85.57	99.90	100	95.47	66.07	44.86	74.21	51.99	99.77	81.71	98.84	65.96	98.76

Proposed deep CNN-LSTM with self-attention model was compared with LSTM-CNN from Lyu *et al.* [53] and CNN-LSTM [54] under the same experimental scenario in order to further verify the model performance. All results were carefully verified to ensure that the results of the comparison were fair and uniform. The evaluation results for the above profound models are shown in Table 9. The deep CNN-LSTM with self-attention has significantly increased by about 3% for the MHEALTH dataset, compared to LSTM-CNN model of Lyu *et al*. It can also be observed that CNN-LSTM with self-attention outperforms the CNN-LSTM, CNN, and Res-LSTM model, proposed by Mutegeki *et al.* [55], Cruciani *et al.* [56], Ullah *et al.* [57], and Yu Zhao *et al.* [58] on the UCI-HAR by nearly 1%.

**TABLE 9:** A Comparative Analysis of the Proposed Model with Respect to the Accuracy of Existing Literature Work

Dataset	Architecture	Accuracy	Year	Ref.
MHEALTH	LSTM-CNN	95.56	2017	Lyu et al. [53]
	CNN,LSTM	93.80	2016	Ordóñez et al. [54]
–	CNN-LSTM with Self-Attention Model(M4)	98.76	–	–Proposed
UCI-HAR	CNN-LSTM	92	2020	Mutegeki et al [55]
	CNN	91.98	2020	Cruciani et al [56]
	Stacked LSTM	93	2019	Ullah et al [57]
	Res-LSTM	91.6	2018	Yu Zhao et al [58]
–	CNN-LSTM with Self-Attention Model(M4)	93.11	–	– Proposed

## VII. Discussion

In this section we conducted a series of experiments to extensively assess the effectiveness of the model described above in order to confirm that the Deep CNN-LSTM with Self-Attention model would perform as expected. We used the three datasets discussed in Section III above to run various experiments on the models, and the results are shown in the following sections.

Initially, data has been categorized into different classes to make classification easier, i.e., sitting, walking, jogging, running, standing and so on. Different deep learning architectures were designed and tested to find out the best fitted model for the recognition. A parallel dense network has been utilized before output parameters. This parallel processing allows an optimal path to be chosen in the hidden layer. Residual connection from the previous layer also prevents vanishing gradient problems [52]. The findings of the study clearly state that various forms of activity can be easily identified.

We demonstrate how convolution processes are robust enough to be applied directly to raw sensor data to extract features that surpass earlier results on the subject within a deep framework. The use of CNNs has the advantage of avoiding hand-crafted or heuristic features, which reduces engineering bias. This is especially crucial when using activity recognition approaches in domains with more complicated activities or open-ended scenarios, where classifiers must adapt to a changing number of classes.

**FIGURE 8.**
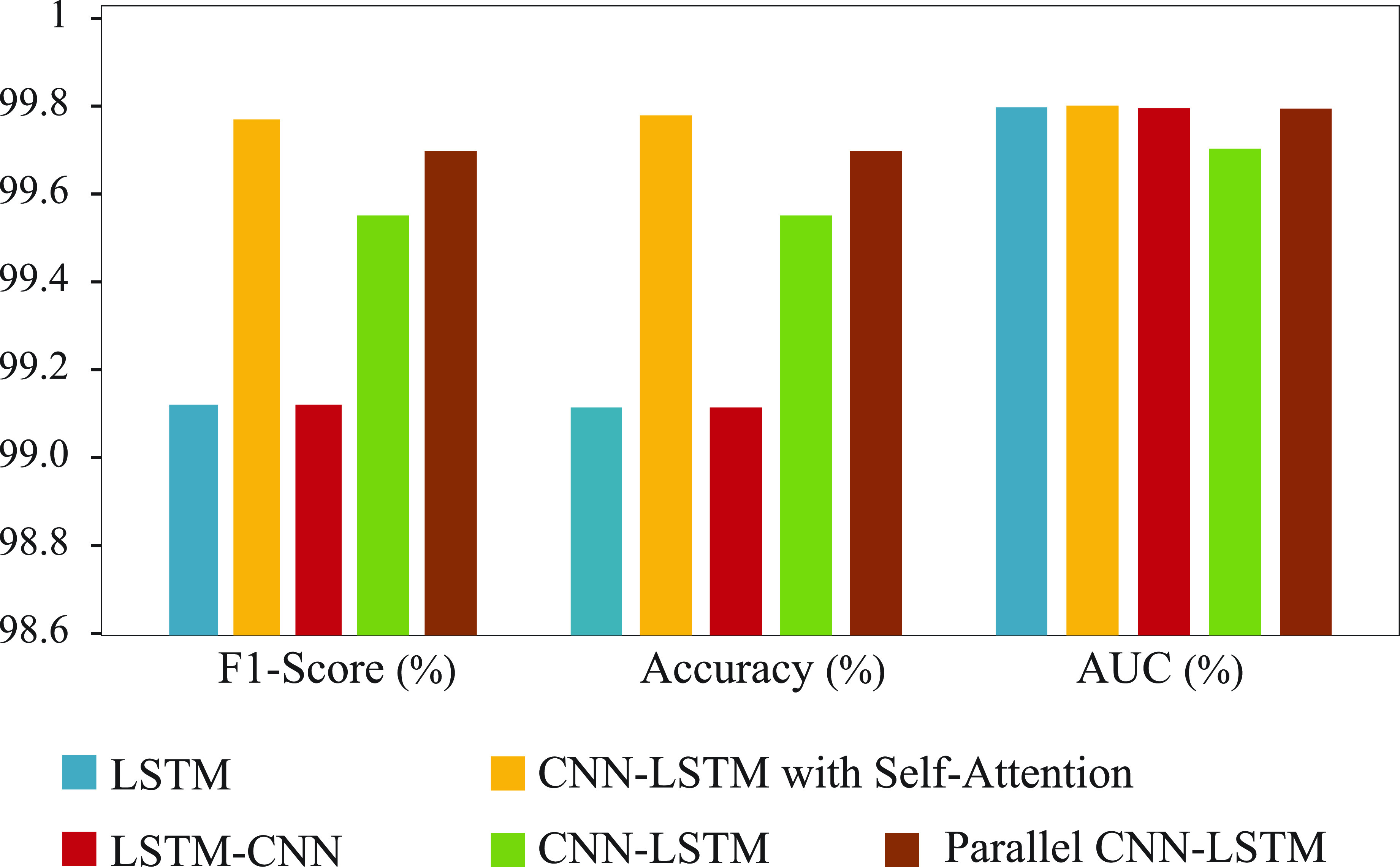
Performance comparison of different (LSTM, CNN-LSTM with Self-Attention, LSTM-CNN, and Parallel CNN-LSTM) models.

We used the deep CNN-LSTM with self-attention model, which is a novel model to be used in the research problems like this. Three different models (i.e., M1, M2, and M3) have been compared with our chosen model (i.e., M4). In contrast to these three models, i.e.,sequential LSTM layers **(LSTM-CNN**) before Convolution layer [48], sequential Convolution and dropout layers **(CNN-LSTM)** before LSTM layer [59], parallel LSTM layers **(Parrallel LSTM-CNN)** with Convolution layer [60], our chosen model achieved the best testing result of 99.93% for H-Activity dataset and 98.76% and 93.11% for MHEALTH and UCI-HAR dataset respectively. Figure 8 compares test F1-Score, Accuracy and AUC among these models. Note, the comparison using final statistical measures with other existing approaches was not conducted since the data set and experimental settings differ from our article.

Hence, all of the above-mentioned models [48], [59], [60] were implemented with exact parameters described in the respective literature except the input and output layer to match our dataset. F1-Score, Accuracy and AUC of best performing Attention CNN-LSTM model **(Model4)** from the previous sub-section has been added in the figure 6 -C for contrast. Although every model has achieved sufficient performance in testing data, CNN-LSTM with self-attention model has better results than other models. Thus the significance of the deep CNN-LSTM with self-attention model has been proved for the human activity recognition approach.

## VIII. Conclusion

We have evaluated a Deep CNN-LSTM with Self-Attention model using Wearable Sensor for the classification of daily activities. We presented this network model using raw accelerometers, gyroscopes and linear Acceleration Data of a smartphone for the input. We also used two benchmark datasets, i.e MHEALTH and UCI-HAR to demonstrate the robustness of our proposed model and get accuracy 98.76% and 93.11% for MHEALTH and UCI-HAR datasets respectively. Our research shows how the automated feature engine in CNN and LSTMs can efficiently extract these characteristics. In a four-class activity recognition scenario with a ten voluntary personalized data set, i.e. H-Activity, the presented model achieved an accuracy of 99.93%. The proposed model demonstrated greater solidity and was more likely than models using statistical machine learning techniques to detect human activity. In the future, we will first continue to strengthen our dataset by adding more participants and adjusting our network structure. Our future research will concentrate on real-time classification of elderly health issues and security systems. We will also focus on the development of wearable and phone based tracking system. Thus, we believe that this developed framework could be applicable in the clinical setting and collected data could be useful for further research.

## References

[1] R. Abdel-Salam, R. Mostafa, and M. Hadhood, Human Activity Recognition Using Wearable Sensors: Review, Challenges, Evaluation Benchmark. Singapore: Springer, Feb. 2021, pp. 1–15.

[2] C. Torres-Huitzil and A. Alvarez-Landero, Accelerometer-Based Human Activity Recognition in Smartphones for Healthcare Services. Cham, Switzerland: Springer, 2015, pp. 147–169, doi: 10.1007/978-3-319-12817-7_7.

[3] J. Wang, Y. Chen, S. Hao, X. Peng, and L. Hu, “Deep learning for sensor-based activity recognition: A survey,” Pattern Recognit. Lett., vol. 119, pp. 3–11, Mar. 2019, doi: 10.1016/j.patrec.2018.02.010.

[4] C. K. Wong, H. M. Mentis, and R. Kuber, “The bit doesn’t fit: Evaluation of a commercial activity-tracker at slower walking speeds,” Gait Posture, vol. 59, pp. 177–181, Jan. 2018, doi: 10.1016/j.gaitpost.2017.10.010. 29049964

[5] A. Murad and J.-Y. Pyun, “Deep recurrent neural networks for human activity recognition,” Sensors, vol. 17, no. 11, p. 2556, Nov. 2017. [Online]. Available: https://www.mdpi.com/1424-8220/17/11/2556 10.3390/s17112556 PMC5712979 29113103

[6] F. Ordóñez and D. Roggen, “Deep convolutional and LSTM recurrent neural networks for multimodal wearable activity recognition,” Sensors, vol. 16, no. 1, p. 115, Jan. 2016. [Online]. Available: https://www.mdpi.com/1424-8220/16/1/115 10.3390/s16010115 PMC4732148 26797612

[7] Y. Guan and T. Plötz, “Ensembles of deep LSTM learners for activity recognition using wearables,” Proc. ACM Interact., Mobile, Wearable Ubiquitous Technol., vol. 1, no. 2, pp. 1–28, 2017, doi: 10.1145/3090076.

[8] H. F. Nweke, Y. W. Teh, M. A. Al-Garadi, and U. R. Alo, “Deep learning algorithms for human activity recognition using mobile and wearable sensor networks: State of the art and research challenges,” Expert Syst. Appl., vol. 105, pp. 233–261, Sep. 2018. [Online]. Available: https://www.sciencedirect.com/science/article/pii/S0957417418302136

[9] O. D. Lara and M. A. Labrador, “A survey on human activity recognition using wearable sensors,” IEEE Commun. Surveys Tuts., vol. 15, no. 3, pp. 1192–1209, Jul. 2013.

[10] M. Shoaib, S. Bosch, O. D. Incel, H. Scholten, and P. Havinga, “A survey of online activity recognition using mobile phones,” Sensors, vol. 15, no. 1, pp. 2059–2085, Jan. 2015. [Online]. Available: https://www.mdpi.com/1424-8220/15/1/2059 25608213 10.3390/s150102059 PMC4327117

[11] J.-B. Yang, N. Nhut, P. San, X. li, and P. Shonali, “Deep convolutional neural networks on multichannel time series for human activity recognition,” in Proc. IJCAI, Jul. 2015, pp. 3995–4001.

[12] C. A. Ronao and S.-B. Cho, “Human activity recognition with smartphone sensors using deep learning neural networks,” Expert Syst. Appl., vol. 59, pp. 235–244, Oct. 2016. [Online]. Available: https://www.sciencedirect.com/science/article/pii/S0957417416302056

[13] J.-H. Hong, J. Ramos, and A. K. Dey, “Toward personalized activity recognition systems with a semipopulation approach,” IEEE Trans. Human-Mach. Syst., vol. 46, no. 1, pp. 101–112, Feb. 2016.

[14] R. Igual, C. Medrano, and I. Plaza, “A comparison of public datasets for acceleration-based fall detection,” Med. Eng. Phys., vol. 37, no. 9, pp. 870–878, Sep. 2015. [Online]. Available: https://www.sciencedirect.com/science/article/pii/S1350453315001575 26233258 10.1016/j.medengphy.2015.06.009

[15] C. Medrano, R. Igual, I. Plaza, and M. Castro, “Detecting falls as novelties in acceleration patterns acquired with smartphones,” PLoS ONE, vol. 9, no. 4, Apr. 2014, Art. no. e94811. 10.1371/journal.pone.0094811 PMC3988107 24736626

[16] F. BagaláÃČÂĆÃÂă, “Evaluation of accelerometer-based fall detection algorithms on real-world falls,” PLoS ONE, vol. 7, no. 5, May 2012, Art. no. e37062. 10.1371/journal.pone.0037062 PMC3353905 22615890

[17] N. D. Lane, “Enabling large-scale human activity inference on smartphones using community similarity networks (CSN),” in Proc. 13th Int. Conf. Ubiquitous Comput. (UbiComp), 2011, pp. 355–364.

[18] G. Weiss and J. Lockhart, “The impact of personalization on smartphone-based activity recognition,” in Proc. AAAI Workshop, Jan. 2012.

[19] M. Shoaib, S. Bosch, O. D. Incel, H. Scholten, and P. J. M. Havinga, “Fusion of smartphone motion sensors for physical activity recognition,” Sensors, vol. 14, no. 6, pp. 10146–10176, 2014. [Online]. Available: https://www.mdpi.com/1424-8220/14/6/10146 24919015 10.3390/s140610146 PMC4118351

[20] M. D. Oresti Banos and R. Garcia, “A novel framework for agile development of mobile health applications,” in Proc. IWAAL, vol. 8868, 2014, pp. 91–98.

[21] O. Banos, “Design, implementation and validation of a novel open framework for agile development of mobile health applications,” Biomed. Eng. OnLine, vol. 14, no. 2, p. S6, 2015. 10.1186/1475-925X-14-S2-S6 PMC4547155 26329639

[22] D. Anguita, A. Ghio, L. Oneto, X. Parra, and J. Reyes-Ortiz, “A public domain dataset for human activity recognition using smartphones,” in Proc. 21st Int. Eur. Symp. Artif. Neural Netw., Comput. Intell. Mach. Learn., Jan. 2013, pp. 437–442.

[23] F. Juefei-Xu, C. Bhagavatula, A. Jaech, U. Prasad, and M. Savvides, “Gait-ID on the move: Pace independent human identification using cell phone accelerometer dynamics,” in Proc. IEEE 5th Int. Conf. Biometrics, Theory, Appl. Syst. (BTAS), Sep. 2012, pp. 8–15.

[24] J. R. Kwapisz, G. M. Weiss, and S. A. Moore, “Cell phone-based biometric identification,” in Proc. 4th IEEE Int. Conf. Biometrics, Theory, Appl. Syst. (BTAS), Sep. 2010, pp. 1–7.

[25] B. Sun, Y. Wang, and J. Banda, “Gait characteristic analysis and identification based on the iPhone’s accelerometer and gyrometer,” Sensors, vol. 14, no. 9, pp. 17037–17054, Sep. 2014. 25222034 10.3390/s140917037 PMC4208212

[26] J. Le Moing and I. Stengel, “The smartphone as a gait recognition device impact of selected parameters on gait recognition,” in Proc. Int. Conf. Inf. Syst. Secur. Privacy (ICISSP), 2015, pp. 322–328.

[27] H. Abujrida, E. Agu, and K. Pahlavan, “Smartphone-based gait assessment to infer Parkinson’s disease severity using crowdsourced data,” in Proc. IEEE Healthcare Innov. Point Care Technol. (HI-POCT), Nov. 2017, pp. 208–211.

[28] J. Juen, Q. Cheng, V. Prieto-Centurion, J. A. Krishnan, and B. Schatz, “Health monitors for chronic disease by gait analysis with mobile phones,” Telemedicine e-Health, vol. 20, no. 11, pp. 1035–1041, Nov. 2014. 24694291 10.1089/tmj.2014.0025 PMC4229704

[29] Y. Ren, Y. Chen, M. C. Chuah, and J. Yang, “User verification leveraging gait recognition for smartphone enabled mobile healthcare systems,” IEEE Trans. Mobile Comput., vol. 14, no. 9, pp. 1961–1974, Sep. 2014.

[30] M. Muaaz and R. Mayrhofer, “Smartphone-based gait recognition: From authentication to imitation,” IEEE Trans. Mobile Comput., vol. 16, no. 11, pp. 3209–3221, Nov. 2017.

[31] F. Demrozi, G. Pravadelli, A. Bihorac, and P. Rashidi, “Human activity recognition using inertial, physiological and environmental sensors: A comprehensive survey,” IEEE Access, vol. 8, pp. 210816–210836, 2020. 33344100 10.1109/access.2020.3037715 PMC7748247

[32] S. Ha and S. Choi, “Convolutional neural networks for human activity recognition using multiple accelerometer and gyroscope sensors,” in Proc. Int. Joint Conf. Neural Netw. (IJCNN), Jul. 2016, pp. 381–388.

[33] S. Dhanraj, S. De, and D. Dash, “Efficient smartphone-based human activity recognition using convolutional neural network,” in Proc. Int. Conf. Inf. Technol. (ICIT), Dec. 2019, pp. 307–312.

[34] V. Bijalwan, V. B. Semwal, G. Singh, and T. K. Mandal, “HDL-PSR: Modelling spatio-temporal features using hybrid deep learning approach for post-stroke rehabilitation,” Neural Process. Lett., vol. 54, no. 3, pp. 1–20, Jan. 2022.

[35] Y. Liu, “A long short-term memory-based model for greenhouse climate prediction,” Int. J. Intell. Syst., vol. 37, no. 1, pp. 135–151, 2022.

[36] Q. Liu, “A fully connected deep learning approach to upper limb gesture recognition in a secure FES rehabilitation environment,” Int. J. Intell. Syst., vol. 36, no. 5, pp. 2387–2411, May 2021.

[37] Y. Liu, “An attention-based category-aware GRU model for the next POI recommendation,” Int. J. Intell. Syst., vol. 36, no. 7, pp. 3174–3189, Jul. 2021.

[38] P. Patil, K. S. Kumar, N. Gaud, and V. B. Semwal, “Clinical human gait classification: Extreme learning machine approach,” in Proc. 1st Int. Conf. Adv. Sci., Eng. Robot. Technol. (ICASERT), May 2019, pp. 1–6.

[39] V. B. Semwal, P. Lalwani, M. K. Mishra, V. Bijalwan, and J. S. Chadha, “An optimized feature selection using bio-geography optimization technique for human walking activities recognition,” Computing, vol. 103, no. 12, pp. 2893–2914, Dec. 2021.

[40] V. B. Semwal, A. Gupta, and P. Lalwani, “An optimized hybrid deep learning model using ensemble learning approach for human walking activities recognition,” J. Supercomput., vol. 77, pp. 12256–12279, Apr. 2021.

[41] V. Bijalwan, V. B. Semwal, and T. K. Mandal, “Fusion of multi-sensor-based biomechanical gait analysis using vision and wearable sensor,” IEEE Sensors J., vol. 21, no. 13, pp. 14213–14220, Jul. 2021.

[42] M. N. Malik, M. A. Azam, M. Ehatisham-Ul-Haq, W. Ejaz, and A. Khalid, “ADLAuth: Passive authentication based on activity of daily living using heterogeneous sensing in smart cities,” Sensors, vol. 19, no. 11, p. 2466, May 2019. 10.3390/s19112466 PMC6603561 31146477

[43] R. Jain, V. B. Semwal, and P. Kaushik, “Deep ensemble learning approach for lower extremity activities recognition using wearable sensors,” Expert Syst., Jun. 2021, Art. no. e12743.

[44] Sensors Data Collector—Apps on Google Play. Accessed: Feb. 23rd, 2020. [Online]. Available: https://play.google.com/store/apps/details?id=com.opendata.labs.sensorsdatacollector

[45] J. R. Kwapisz, G. M. Weiss, and S. A. Moore, “Activity recognition using cell phone accelerometers,” ACM SIGKDD Explor. Newslett., vol. 12, no. 2, pp. 74–82, May 2011.

[46] A. Bayat, M. Pomplun, and D. A. Tran, “A study on human activity recognition using accelerometer data from smartphones,” Proc. Comput. Sci., vol. 34, pp. 450–457, Jan. 2014.

[47] M. N. Noor, A. S. Yahaya, N. A. Ramli, and A. M. M. Al Bakri, “Filling missing data using interpolation methods: Study on the effect of fitting distribution,” Key Eng. Mater., vols. 594–595, pp. 889–895, Dec. 2013.

[48] K. Xia, J. Huang, and H. Wang, “LSTM-CNN architecture for human activity recognition,” IEEE Access, vol. 8, pp. 56855–56866, 2020.

[49] A. Krizhevsky, I. Sutskever, and G. E. Hinton, “Imagenet classification with deep convolutional neural networks,” in Proc. Adv. Neural Inf. Process. Syst., vol. 25, F. Pereira, C. J. C. Burges, L. Bottou, and K. Q. Weinberger, Eds. Red Hook, NY, USA: Curran Associates, 2012. [Online]. Available: https://proceedings.neurips.cc/paper/2012/file/c399862d3b9d6b76c8436e924a68c45b-Paper.pdf

[50] Y. Bengio, P. Simard, and P. Frasconi, “Learning long-term dependencies with gradient descent is difficult,” IEEE Trans. Neural Netw., vol. 5, no. 2, pp. 157–166, Mar. 1994. 18267787 10.1109/72.279181

[51] S. Hochreiter and J. Schmidhuber, “Long short-term memory,” Neural Comput., vol. 9, no. 8, pp. 1735–1780, 1997. 9377276 10.1162/neco.1997.9.8.1735

[52] Y. Hu, A. Huber, J. Anumula, and S.-C. Liu, “Overcoming the vanishing gradient problem in plain recurrent networks,” 2018, arXiv:1801.06105.

[53] L. Lyu, X. He, Y. W. Law, and M. Palaniswami, “Privacy-preserving collaborative deep learning with application to human activity recognition,” in Proc. Conf. Inf. Knowl. Manage., Nov. 2017, pp. 1219–1228.

[54] D. R. Francisco Javier Ordóñez, “Deep convolutional and lstm recurrent neural networks for multimodal wearable activity recognition,” Sensors, vol. 16, no. 1, p. 115, Jan. 2016. 10.3390/s16010115 PMC4732148 26797612

[55] R. Mutegeki and D. S. Han, “A CNN-LSTM approach to human activity recognition,” in Proc. Int. Conf. Artif. Intell. Inf. Commun., 2020, pp. 362–366.

[56] F. Cruciani, “Feature learning for human activity recognition using convolutional neural networks: A case study for inertial measurement unit and audio data,” CCF Trans. Pervasive Comput. Interact., vol. 2, no. 1, pp. 18–32, Mar. 2020.

[57] M. Ullah, H. Ullah, S. D. Khan, and F. A. Cheikh, “Stacked LSTM network for human activity recognition using smartphone data,” in Proc. 8th Eur. Workshop Vis. Inf. Process. (EUVIP), Oct. 2019, pp. 175–180.

[58] Y. Zhao, R. Yang, G. Chevalier, and M. Gong, “Deep residual bidir-LSTM for human activity recognition using wearable sensors,” Math. Problems Eng., vol. 2018, Dec. 2018.

[59] F. Li, K. Shirahama, M. Nisar, L. Köping, and M. Grzegorzek, “Comparison of feature learning methods for human activity recognition using wearable sensors,” Sensors, vol. 18, no. 3, p. 679, Feb. 2018. 10.3390/s18020679 PMC5855052 29495310

[60] Q. Zou, Y. Wang, Q. Wang, Y. Zhao, and Q. Li, “Deep learning-based gait recognition using smartphones in the wild,” IEEE Trans. Inf. Forensics Security, vol. 15, pp. 3197–3212, 2020.

